# Utility of Leptomeningeal Collaterals in Predicting Intracranial Atherosclerosis-Related Large Vessel Occlusion in Endovascular Treatment

**DOI:** 10.3390/jcm9092784

**Published:** 2020-08-28

**Authors:** Jang-Hyun Baek, Byung Moon Kim, Jin Woo Kim, Dong Joon Kim, Ji Hoe Heo, Hyo Suk Nam, Young Dae Kim

**Affiliations:** 1 Department of Neurology, Kangbuk Samsung Hospital, Sungkyunkwan University School of Medicine, Seoul 03181, Korea; janghyun.baek@gmail.com; 2 Department of Neurology, Severance Stroke Center, Severance Hospital, Yonsei University College of Medicine, Seoul 03722, Korea; jhheo@yuhs.ac (J.H.H.); hsnam@yuhs.ac (H.S.N.); neuro05@yuhs.ac (Y.D.K.); 3 Interventional Neuroradiology, Severance Stroke Center, Severance Hospital, Department of Radiology, Yonsei University College of Medicine, Seoul 03722, Korea; djkimmd@yuhs.ac; 4 Department of Radiology, Gangnam Severance Hospital, Yonsei University College of Medicine, Seoul 06273, Korea; sunny-cocktail@hanmail.net

**Keywords:** atherosclerosis, computed tomography angiography, stroke, thrombectomy

## Abstract

Earlier or preprocedural identification of occlusion pathomechanism is crucial for effective endovascular treatment. As leptomeningeal collaterals tend to develop well in chronic ischemic conditions such as intracranial atherosclerosis (ICAS), we investigated whether leptomeningeal collaterals can be a preprocedural marker of ICAS-related large vessel occlusion (ICAS-LVO) in endovascular treatment. A total of 226 patients who underwent endovascular treatment were retrospectively reviewed. We compared the pattern of leptomeningeal collaterals between patients with ICAS-LVO and without. Leptomeningeal collaterals were assessed by preprocedural computed tomography angiography (CTA) and basically categorized by three different collateral assessment methods. Better leptomeningeal collaterals were significantly associated with ICAS-LVO, although they were not independent for ICAS-LVO. When leptomeningeal collaterals were dichotomized to incomplete (<100%) and complete (100%), the latter was significantly more frequent in patients with ICAS-LVO (52.5% versus 20.4%) and remained an independent factor for ICAS-LVO (odds ratio, 3.32; 95% confidence interval, 1.52–7.26; *p* = 0.003). The area under the curve (AUC) value of complete leptomeningeal collateral supply was 0.660 for discrimination of ICAS-LVO. Incomplete leptomeningeal collateral supply was not likely ICAS-LVO, based on the high negative predictive value (88.6%). Considering its negative predictive value and the independent association between complete leptomeningeal collateral supply and ICAS-LVO, leptomeningeal collaterals could be helpful in the preprocedural determination of occlusion pathomechanism.

## 1. Introduction

Mechanical thrombectomy has been primarily considered in most cases of endovascular treatment of acute intracranial large vessel occlusion [1]. However, mechanical thrombectomy might not be an optimal modality for a specific occlusion pathomechanism—that is, an in situ thrombo-occlusion of underlying intracranial atherosclerosis (intracranial atherosclerosis-related large vessel occlusion (ICAS-LVO)) [2,3]. ICAS-LVO is not a rare condition. In the Asian population, up to 30% of patients might have ICAS-LVO for their occlusion pathomechanism in endovascular treatment of anterior circulation [4]. More importantly, conventional mechanical thrombectomy modalities, such as stent retriever and thrombaspiration, are ineffective in ICAS-LVO. Mechanical thrombectomy was effective only in less than 20% of ICAS-LVO cases. Due to frequent reocclusion events, specific rescue endovascular modalities (i.e., intra-arterial glycoprotein IIb/IIIa inhibitor, balloon angioplasty, and intracranial stenting) were inevitable in most cases to achieve significant recanalization in ICAS-LVO [5,6,7,8,9].

On this point, earlier strategical consideration is crucial to shorten the time to recanalization [4]. For earlier strategical consideration, it could be more helpful if the occlusion pathomechanism is determined before endovascular treatment. However, the completion of such a preprocedural determination is challenging as the information available before endovascular treatment can be limited. In clinical practice, we are able to rely on only a few preprocedural clinical and imaging findings [7,10]. However, further reliable methods are sparse.

Leptomeningeal collaterals are one of the preprocedural factors which are potentially able to predict occlusion pathomechanism. Nevertheless, their association has not been clearly evaluated yet. Several experimental and clinical findings led us to focus on leptomeningeal collaterals. In these experimental findings, the vascular bed was more developed in chronic or long-term ischemic conditions [11,12,13]. Similarly, in patients with an intracranial stenosis due to ICAS, leptomeningeal collaterals were prominently developed to compensate for the diminished cerebral blood flow under chronic ischemia [11,12]. In one report, full and rapid leptomeningeal collateral filling was commented on as a finding, which suggests ICAS-LVO [7]. However, no specific evidence supported this comment. Instead, it was reported that initial infarct volume was smaller among patients with an ICAS-LVO. This merely indirectly suggested that leptomeningeal collaterals were better in ICAS-LVO [7,14].

If leptomeningeal collaterals are discriminatorily developed in patients with ICAS, we believe that they may be an indirect finding for ICAS-LVO. Thus, we hypothesized that (1) leptomeningeal collaterals would be different according to the occlusion pathomechanism—that is, robust or better leptomeningeal collaterals are associated with ICAS-LVO, and (2) based on this association, we could predict ICAS-LVO before endovascular treatment. Accordingly, this study aimed to evaluate (1) the association between leptomeningeal collaterals and ICAS-LVO, and (2) the predictability of preprocedural leptomeningeal collaterals for ICAS-LVO.

## 2. Methods

We retrospectively reviewed consecutive acute stroke patients between January 2010 and December 2018 who underwent endovascular treatment of intracranial vessel occlusion. Patients were selected from a prospective registry of a tertiary stroke center (Severance Stroke Center, Severance Hospital, Seoul, Korea). The registry consists of consecutive patients who underwent endovascular treatment, which was considered by the following criteria: (1) a computed tomography angiography (CTA)-determined, endovascularly accessible intracranial LVO associated with neurological symptoms; (2) within 8 h from stroke onset, though, in the later study period, patients falling within the window of 8 h to 12 h from stroke onset were also considered if they had an Alberta Stroke Program Early CT Score of seven points or more on initial non-contrast CT; and (3) a baseline National Institutes of Health Stroke Scale (NIHSS) score of four points or more. For patients eligible for intravenous tissue-type plasminogen activator treatment, the full dose of tissue-type plasminogen activator (0.9 mg/kg) was administered.

For this study, patients who had an M1 occlusion and CTA performed before endovascular treatment were selected from the registry. Conversely, those who presented with an internal carotid artery occlusion were excluded, as collateral flows through anterior or posterior communicating arteries can contribute to lesion-side cerebral flow. Additionally, patients with an occlusion of the distal artery or posterior circulation were also excluded because leptomeningeal collaterals could not be determined reliably in this population. We did not include patients with multiple intracranial artery occlusions because they could also affect leptomeningeal collaterals on middle cerebral artery territory.

The institutional review board approved this study and waived the requirement for obtaining informed consent prior to study inclusion based on the retrospective design.

### 2.1. Assessment of Leptomeningeal Collaterals (Collateral Assessment Methods)

Leptomeningeal collaterals were determined by CTA performed immediately before endovascular treatment. CTA collateral grade was assessed on 20-mm thickness maximum intensity projection images of single-phase CTA. In patients who underwent multiphase CTA imaging, we only used the first-phase images to evaluate leptomeningeal collaterals.

**Figure 1:**
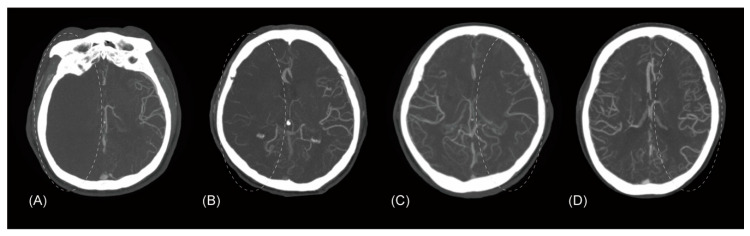
Assessment of leptomeningeal collaterals according to collateral assessment method 1 (Tan et al.’s method). Four computed tomography angiography maximum intensity projection axial images from different patients showing leptomeningeal collaterals. (**A**) Absent collateral supply to the occluded middle cerebral artery territory (circle), compared to the contralateral normal side. (**B**) Collateral supply of greater than 0% but less than or equal to 50%. (**C**) Collateral supply of greater than 50% but less than 100%. (**D**) Complete collateral supply of 100%.

From among the various existing CTA-based collateral assessment methods, three different methods were adopted [15,16,17]. First, leptomeningeal collaterals were primarily assessed by a four-scale method previously reported as follows: (1) absent collateral supply to the occluded middle cerebral artery (MCA) territory of 0%, (2) collateral supply of greater than 0% but less than or equal to 50%, (3) collateral supply of greater than 50% but less than 100%, and (4) complete collateral supply of 100% (collateral assessment method 1; Tan’s method; Figure 1) [15]. Second, the four grades were regrouped into a three-scale grade system as follows: (1) absent collateral supply to the occluded MCA territory of 0%, (2) collateral supply of greater than 0% but less than 100%, and (3) complete collateral supply of 100% (collateral assessment method 2; shortened from Mass’ method) [16]. Third, the four grades were simply dichotomized into (1) collateral supply of 50% or less of the occluded MCA territory and (2) collateral supply of more than 50% (collateral assessment method 3; modified Tan’s method) [17]. The four-scale grade of leptomeningeal collaterals was determined by two independent interventional neuroradiologists who were blinded to the clinical and procedural information. The kappa value for the interrater agreement was 0.85 (95% confidence interval, 0.79–0.91), which was similar to its original report [15]. Discrepant cases were resolved by consensus.

### 2.2. Identification of ICAS-LVO

ICAS-LVO was determined angiographically. If the occlusion site was completely recanalized without any residual stenosis and reocclusion tendency, the occlusion pathomechanism was not considered as ICAS. In contrast, when significant fixed focal stenosis was noted on angiography, the case was considered as positive for ICAS-LVO [7]. For intractable cases whose occlusion was never recanalized, so that the focal stenosis could not be evaluated, occlusion at the arterial trunk was determined as indicative of ICAS-LVO [6]. ICAS-LVO was assessed independently by two other interventional neuroradiologists who were blinded to the CTA findings and clinical information. The kappa value for the interrater agreement was 0.92 (95% confidence interval, 0.86–0.98). Discrepant cases were also resolved by consensus.

### 2.3. Statistical Analysis

Based on the identification of ICAS-LVO, patients were assigned to the ICAS group or the non-ICAS group. First, we evaluated the association between leptomeningeal collaterals as determined by the three collateral assessment methods and ICAS-LVO. In this process, (1) basic demographics (age and sex), risk factors for atherosclerosis (hypertension diabetes, dyslipidemia, smoking, and coronary artery disease), typical clinical factors associated with occlusion pathomechanism (atrial fibrillation and initial NIHSS score), and leptomeningeal collaterals by each collateral assessment method were compared between the ICAS and non-ICAS groups. The Mann–Whitney U test, chi-squared test, and Fisher’s exact test were used for comparison. Also, we summarized the study population by descriptive statistics. Continuous variables were expressed by a mean value with standard deviation or a median value with interquartile range, as appropriate. Categorical variables were expressed by a frequency with its percentage. Then, (2) to see whether better leptomeningeal collaterals were associated with ICAS-LVO, we performed binary logistic regression analyses for each collateral assessment method. To determine whether better leptomeningeal collaterals can be an independent variable for ICAS-LVO, variables with a *p*-value < 0.10 in the univariable analysis were entered into the multivariable model. Finally, (3) to evaluate the predictive power of leptomeningeal collaterals for ICAS-LVO, we calculated the sensitivity, specificity, positive predictive value (PPV), negative predictive value (NPV), and accuracy of each collateral assessment method. Receiver operating characteristic curve analyses were also performed to calculate the area under the curve (AUC) values and cutoff points, which were determined based on Youden’s index.

Second, based on the results of logistic regression analyses and calculated cutoff points of each collateral assessment method, leptomeningeal collaterals were dichotomized into (1) incomplete collateral supply of less than 100% or (2) complete collateral supply of 100%. Then, the findings of complete and incomplete collateral supplies were compared between the ICAS and non-ICAS groups. To see whether complete leptomeningeal collateral supply was associated with ICAS-LVO, univariable and multivariable binary logistic regression analyses were performed in the same manner as the three collateral assessment methods. We also calculated sensitivity, PPV, NPV, accuracy, and AUC value for the dichotomization in predicting ICAS-LVO.

A *p*-value < 0.05 was considered statistically significant for the 95% confidence interval. All statistical analyses were performed using R software (version 3.5.0; R Foundation for Statistical Computing, Vienna, Austria).

## 3. Results

**Figure 2:**
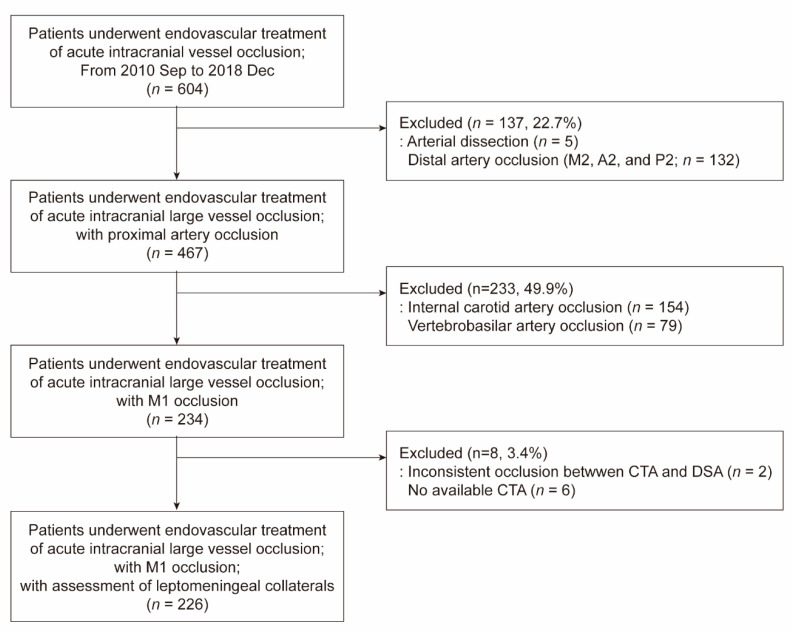
Patients selection flow chart. DSA, digital subtraction angiography.

Among the 604 patients that underwent endovascular treatment for an intracranial vessel occlusion, 226 patients (mean age 69.0 ± 12.1 years; 54.4% male) were included (Figure 2). Patients with arterial dissection (*n* = 5), distal artery occlusion (*n* = 132), internal carotid artery occlusion (*n* = 154), and vertebrobasilar artery occlusion (*n* = 79) were excluded. In eight patients, leptomeningeal collaterals could not be determined by CTA because the arterial target was changed between CTA and endovascular treatment (*n* = 2; internal carotid artery occlusion on initial CTA was changed to M1 occlusion on cerebral angiography) or CTA was not performed before endovascular treatment (*n* = 6). Leptomeningeal collaterals were 0% in the occluded MCA territory in 15 patients (6.6%), greater than 0% but less than or equal to 50% in 57 (25.2%), greater than 50% but less than 100% in 95 (42.1%), and 100% in 59 (26.1%).

### 3.1. Association Between Leptomeningeal Collaterals and ICAS-LVO

A total of 40 patients (17.7%) showed an ICAS-LVO as the occlusion pathomechanism. Based on the use of collateral assessment methods 1 and 2, patients with leptomeningeal collaterals of 0%, greater than 0% but less than or equal to 50%, and greater than 50% but less than 100% of occluded MCA territory were less common in the ICAS group, whereas cases of complete (100%) leptomeningeal collaterals were significantly more frequently found in the ICAS group (52.5% versus 20.4%; *p* < 0.001; Table 1). For collateral assessment method 3, more patients in the ICAS group had leptomeningeal collaterals of greater than 50% than the non-ICAS group (85.0% versus 64.5%; *p* = 0.012).

**Figure 3:**
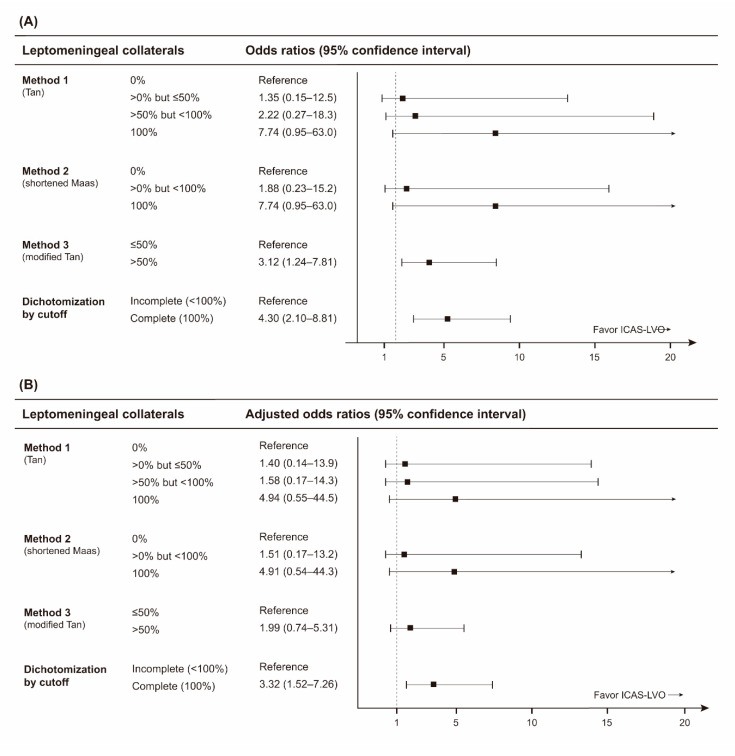
Univariable and multivariable logistic regression analyses of leptomeningeal collaterals for intracranial atherosclerosis-related large vessel occlusion (ICAS-LVO). Odds ratios with 95% confidence intervals from (**A**) univariable and (**B**) multivariable logistic regression analyses are plotted. In multivariable analyses, each leptomeningeal collateral assessment method was adjusted by current smoking, atrial fibrillation, and initial NIHSS score.

On the logistic regression analyses for collateral assessment methods 1, 2, and 3, odds ratios for ICAS-LVO gradually increased as leptomeningeal collaterals improved (Figure 3). However, none were statistically significant in univariable and multivariable analyses (Table S1 and Figure 3). For multivariable analyses, each collateral assessment method was adjusted by current smoking, atrial fibrillation, and initial NIHSS score.

### 3.2. Predictive Power of Leptomeningeal Collaterals for ICAS-LVO

For collateral assessment methods 1 and 2, the calculated sensitivity, specificity, PPV, and NPV of leptomeningeal collaterals to predict ICAS-LVO were 52.5%, 79.6%, 35.6%, and 88.6%, respectively (Table 2). Collateral assessment method 3 showed higher sensitivity and lower specificity for ICAS-LVO than collateral assessment methods 1 and 2. AUC values of leptomeningeal collaterals were below 0.7 for all collateral assessment methods (each *p* < 0.001).

### 3.3. Significance of Complete Leptomeningeal Collaterals in Predicting ICAS-LVO

Based on the results from the analyses of collateral assessment methods 1, 2, and 3, study participants’ leptomeningeal collaterals were dichotomized to incomplete (less than 100%) and complete (100%). Patients in the ICAS group showed more complete leptomeningeal collateral supply (*p* < 0.001; Table 1). During multivariable analysis, complete leptomeningeal collateral supply remained an independent factor for ICAS-LVO (odds ratio, 3.32; 95% confidence interval, 1.52–7.26; *p* = 0.003; Table S1 and Figure 3). An AUC value for complete leptomeningeal collaterals was 0.660 (95% CI, 0.595–0.722; *p* < 0.001), with an NPV of 88.6% (Table 2).

## 4. Discussion

In this study, we found that leptomeningeal collaterals were associated with occlusion pathomechanism. Better leptomeningeal collaterals were significantly associated with ICAS-LVO in common collateral assessment methods. Nevertheless, during logistic regression analyses, only the use of a dichotomization method (incomplete versus complete) was independently associated with ICAS-LVO. Despite the independence, it achieved only a modest degree of predictability of ICAS-LVO. To the best of our knowledge, this study is the first study to evaluate the association of leptomeningeal collaterals with ICAS-LVO and its preprocedural possibility to predict ICAS-LVO.

This study was originally contrived from the necessity to enhance the preprocedural determination of occlusion pathomechanism in endovascular treatment of LVO. Because the optimal endovascular strategy—which includes selection of the most effective endovascular modality and when to switch from one modality to another—depends on the type of occlusion pathomechanism, the earlier determination of occlusion pathomechanism can be crucial in attaining significant recanalization [18,19]. However, unfortunately, there have been only a few practical factors identified that we can rely on to identify occlusion pathomechanism before an endovascular procedure. Common cardioembolic sources such as atrial fibrillation or valvular heart diseases are typically considered as evidence of an embolic occlusion of an intracranial artery [7]. Specific imaging findings—for example, a hyperdense artery sign on CT or a blooming artifact on magnetic resonance imaging—have also been regarded as markers of embolic occlusion [14,20]. Uniquely, the occlusion type observed on preprocedural CTA was significantly associated with occlusion pathomechanism. The occlusion type was superior to the atrial fibrillation and hyperdense artery sign in predicting the occlusion pathomechanism [10]. Patient demographics or a few risk factors for atherosclerosis can be referred to in order to assume the occlusion pathomechanism; however, they are quite indirect means [7]. Particular infarct patterns could also be helpful in determining the occlusion pathomechanism. However, the infarct pattern is less evident and may be limited in the preprocedural condition [14].

As we expected, leptomeningeal collaterals were significantly associated with occlusion pathomechanism in this study. In particular, complete leptomeningeal collaterals were consistently associated with ICAS-LVO with a modest level of predictability. In comparison with other preprocedural findings used to predict ICAS-LVO, the discriminative power of complete leptomeningeal collaterals seemed not so inferior. In this study population, the AUC value of complete leptomeningeal collaterals for ICAS-LVO was not lower than that of atrial fibrillation (0.653; 95% confidence interval, 0.587–0.715). Furthermore, the AUC value of CTA-determined occlusion type for stent retriever success, one of the preprocedural findings presumed to be highly associated with ICAS-LVO, was less than 0.7 [5].

To the best of our knowledge, only one other study has commented on the association between leptomeningeal collaterals and ICAS-LVO in endovascular treatment [7]. In the literature, full and rapid leptomeningeal collaterals were significantly more frequent in patients with ICAS-LVO, which was consistent with our study. However, the result was unofficial from a small number of patients, so it was merely a piece of unpublished data in a review article. Additionally, leptomeningeal collaterals were not considered as a predictor of ICAS-LVO in the literature. Leptomeningeal collaterals were assessed by initial cerebral angiogram during endovascular treatment, not preprocedurally.

Complete leptomeningeal collaterals showed low sensitivity and PPV with a relatively higher NPV in discriminating ICAS-LVO. For practical use, the statistical parameters can be interpreted as follows: (1) in patients with ICAS-LVO, the probability of showing complete leptomeningeal collaterals was about 50% (low sensitivity); (2) even among cases showing complete leptomeningeal collaterals, ICAS-LVO was only present in about 40% of them (low PPV); and (3) if a patient showed incomplete leptomeningeal collaterals, occlusion pathomechanism was not likely to be ICAS-LVO up to 90% (high NPV).

The study results might be substantially affected by the chosen collateral assessment method. However, there has been no consensual grading system established to evaluate leptomeningeal collaterals on CTA. To avoid arbitrary grading, we tried to choose collateral assessment methods that have all been widely used in previous studies [21,22]. Based on the consistent findings from those collateral assessment methods, we regrouped the leptomeningeal collaterals by its cutoff value into incomplete or complete. Cerebral angiography can be a good modality with which to assess leptomeningeal collaterals. However, in most endovascular procedures, initial leptomeningeal collateral flow is not assessed on cerebral angiography because the arterial target is directly approached without taking the angiography of other cerebral vessels. We think that CTA might be the most rational modality to use to assess leptomeningeal collaterals in daily clinical practice. Multiphase CTA could be another collateral assessment method. However, multiphase CTA cannot be deployed in all centers; indeed, multiphase CTA was not performed in the early period of this study.

This study had a few limitations. First, it was performed retrospectively in a single tertiary stroke center. However, all patients were prospectively registered with a detailed description of their endovascular procedure. Furthermore, this study focused on objective findings, including imaging markers and angiographic findings rather than on clinical outcomes, thereby minimizing this limitation. Nevertheless, based on the retrospective nature of this study, there might be a possibility that the patients with better leptomeningeal collaterals were preferentially chosen for endovascular treatment. Although the predetermined protocol in our center did not regulate the leptomeningeal collateral status for endovascular treatment eligibility, the physician’s clinical decision might be partly affected by the leptomeningeal collateral status.

Second, this study included patients only with M1 occlusion. Thus, the generalization of our study results to all anterior circulation strokes might be inappropriate. However, as described earlier, such use of this strict inclusion criterion was to ensure the improved evaluation of uncontaminated leptomeningeal collaterals. Study findings should be understood as providing verification of a general hypothesis that better leptomeningeal collaterals are associated with ICAS-LVO. In addition, generalization of the study results could also be limited because this study was performed in an Asian country where ICAS is more prevalent. As statistical power might be affected by the number of patients with ICAS-LVO, no one could precisely figure out the association of leptomeningeal collaterals with ICAS-LVO in other countries where ICAS is much less prevalent.

Third, this study also included patients with a tandem occlusion (M1 occlusion with cervical ICA occlusion/stenosis). Chronic ischemia, even due to severe cervical ICA stenosis, might be associated with robust leptomeningeal collaterals. Thus, theoretically, for the tandem occlusion, leptomeningeal collaterals could be abundant or complete, although its M1 occlusion is embolic from a cervical ICA lesion. In fact, about 5% patients of this study had an atherosclerotic cervical ICA occlusion. Fortunately, even after excluding the patients with tandem occlusions, the significance of the study results was not changed.

## 5. Conclusions

Leptomeningeal collaterals determined by preprocedural CTA were significantly associated with occlusion pathomechanism. Specifically, complete leptomeningeal collateral supply was independently associated with ICAS-LVO. Despite the association, however, leptomeningeal collaterals were simply predictive of ICAS-LVO in a modest degree. In clinical practice, one could assume that incomplete leptomeningeal collateral supply is not likely ICAS-LVO based on high NPV. In this way, leptomeningeal collaterals could be helpful in the preprocedural determination of occlusion pathomechanism.

## Figures and Tables

**Table 1:** Comparison of demographics, risk factors for stroke and atherosclerosis, and leptomeningeal collaterals between intracranial atherosclerosis (ICAS) and non-ICAS groups.

	All (*n* = 226)	ICAS (*n* = 40)	Non-ICAS (*n* = 186)	*p*-Value
Age, years	69.0 (±12.1)	66.2 (±16.4)	69.6 (±10.9)	0.222
Male sex	123 (54.4)	20 (50.0)	103 (55.4)	0.536
Hypertension	166 (73.5)	32 (80.0)	134 (72.0)	0.301
Diabetes	66 (29.2)	13 (32.5)	53 (28.5)	0.613
Dyslipidemia	48 (21.2)	12 (30.0)	36 (19.4)	0.135
Current smoking	42 (18.6)	16 (40.0)	26 (14.0)	<0.001
Coronary artery disease	52 (23.0)	11 (27.5)	41 (22.0)	0.457
Atrial fibrillation	119 (52.7)	11 (27.5)	108 (58.1)	<0.001
Initial NIHSS score	15.0 (11.0; 19.0)	12.0 (7.0; 17.0)	15.0 (12.0; 19.0)	0.001
Leptomeningeal collaterals				
Three assessment methods				
Method 1 (Tan)				
0%	15 (6.6)	1 (2.5)	14 (7.5)	<0.001
>0% but ≤50%	57 (25.2)	5 (12.5)	52 (28.0)	
>50% but <100%	95 (42.1)	13 (32.5)	82 (44.1)	
100%	59 (26.1)	21 (52.5)	38 (20.4)	
Method 2 (shortened Maas)				
0%	15 (6.6)	1 (2.5)	14 (7.5)	<0.001
>0% but <100%	152 (67.3)	18 (45.0)	134 (72.1)	
100%	59 (26.1)	21 (52.5)	38 (20.4)	
Method 3 (modified Tan)				
≤50%	72 (31.9)	6 (15.0)	66 (35.5)	0.012
>50%	154 (68.1)	34 (85.0)	120 (64.5)	
Dichotomization by cutoff				
Incomplete (<100%)	167 (73.9)	19 (47.5)	148 (79.6)	<0.001
Complete (100%)	59 (26.1)	21 (52.5)	38 (20.4)	

Age is represented by a mean value (±standard deviation); initial National Institutes of Health Stroke Scale (NIHSS) score by a median value (first and third quartile); all other variables by the number of patients (frequency, %).

**Table 2:** Diagnostic performance of leptomeningeal collaterals for intracranial atherosclerosis-related large vessel occlusion.

Collateral Assessment Methods	Sensitivity (%)	Specificity (%)	PPV (%)	NPV (%)	Accuracy (%)	AUC
Method 1 (Tan) ^1^	52.5	79.6	35.6	88.6	74.8	0.686
Method 2 (shortened Maas) ^1^	52.5	79.6	35.6	88.6	74.8	0.668
Method 3 (modified Tan) ^2^	85.0	35.5	22.1	91.7	44.2	0.602
Dichotomization by cutoff ^1^	52.5	79.6	35.6	88.6	74.8	0.660

^1^ For leptomeningeal collaterals of 100%; ^2^ for leptomeningeal collaterals of > 50%; PPV, positive predictive value; NPV, negative predictive value; AUC, area under curve.

## Supplementary Materials

The following are available online at https://www.mdpi.com/2077-0383/9/9/2784/s1, Table S1: Multivariable analyses for the association with intracranial atherosclerosis-related large vessel occlusion.

## References

[1] Powers W.J., Rabinstein A.A., Ackerson T., Adeoye O.M., Bambakidis N.C., Becker K., Biller J., Brown M., Demaerschalk B.M., Hoh B. (2019). Guidelines for the early management of patients with acute Ischemic stroke: 2019 Update to the 2018 guidelines for the early management of acute ischemic stroke: A auideline for healthcare professionals from the American Heart Association/American Stroke Association. Stroke.

[2] Kang D.-H., Yoon W., Baek B.H., Kim S.K., Lee Y.Y., Kim J.-T., Park M.-S., Kim Y.-W., Hwang Y.-H., Kim Y.-S. (2020). Front-line thrombectomy for acute large-vessel occlusion with underlying severe intracranial stenosis: Stent retriever versus contact aspiration. J. Neurosurg..

[3] Lee J.S., Lee S.-J., Hong J.M., Choi J.W., Yoo J., Hong J.-H., Kim C.-H., Kim Y.-W., Kang D.-H., Hwang Y.-H. (2018). Solitaire thrombectomy for acute stroke due to intracranial atherosclerosis-related occlusion: ROSE ASSIST study. Front. Neurol..

[4] Tsang A.C.O., Orru E., Klostranec J.M., Yang I.-H., Lau K.K., Tsang F.C.P., Lui W.M., Pereira V.M., Krings T. (2019). Thrombectomy outcomes of intracranial atherosclerosis-related occlusions. Stroke.

[5] Baek J.-H., Kim B., Heo J.H., Nam H.S., Song D., Bang O.Y., Kim D.J. (2016). Importance of truncal-type occlusion in stentriever-based thrombectomy for acute stroke. Neurology.

[6] Baek J.-H., Kim B., Heo J.H., Kim D.J., Nam H.S., Kim Y.D. (2018). Outcomes of endovascular treatment for acute intracranial atherosclerosis–related large vessel occlusion. Stroke.

[7] Lee J.S., Hong J.M., Kim J.S. (2017). Diagnostic and therapeutic strategies for acute intracranial atherosclerosis-related occlusions. J. Stroke.

[8] Park H., Baek J.-H., Kim B. (2019). Endovascular treatment of acute stroke due to intracranial atherosclerotic stenosis-related large vessel occlusion. Front. Neurol..

[9] Kang D.-H., Yoon W. (2019). Current opinion on endovascular therapy for emergent large vessel occlusion due to underlying intracranial atherosclerotic stenosis. Korean J. Radiol..

[10] Baek J.-H., Kim B., Yoo J., Nam H.S., Kim Y.D., Kim D.J., Heo J.H., Bang O.Y. (2017). Predictive value of computed tomography angiography-determined occlusion type in stent retriever thrombectomy. Stroke.

[11] Brozici M., Van Der Zwan A., Hillen B. (2003). Anatomy and functionality of leptomeningeal anastomoses: A review. Stroke.

[12] Shuaib A., Butcher K., A Mohammad A., Saqqur M., Liebeskind D.S. (2011). Collateral blood vessels in acute ischaemic stroke: A potential therapeutic target. Lancet Neurol..

[13] Liebeskind D.S. (2003). Collateral circulation. Stroke.

[14] Suh H.I., Hong J.M., Lee K.S., Han M., Choi J.W., Kim J.S., Demchuk A.M., Lee J.S. (2016). Imaging predictors for atherosclerosis-related intracranial large artery occlusions in acute anterior circulation stroke. J. Stroke.

[15] Tan I., Demchuk A., Hopyan J., Zhang L., Gladstone D.J., Wong K.-K., Martin M., Symons S., Fox A., Aviv R. (2009). CT angiography clot burden score and collateral score: Correlation with clinical and radiologic outcomes in acute middle cerebral artery infarct. AJNR Am. J. Neuroradiol..

[16] Maas M.B., Lev M.H., Ay H., Singhal A.B., Greer D.M., Smith W.S., Harris G.J., Halpern E., Kemmling A., Koroshetz W.J. (2009). Collateral vessels on CT angiography predict outcome in acute ischemic stroke. Stroke.

[17] Kim B., Baek J.-H., Heo J.H., Nam H.S., Kim Y.D., Yoo J., Kim D.J., Jeon P., Baik S.K., Suh S. (2018). Collateral status affects the onset-to-reperfusion time window for good outcome. J. Neurol. Neurosurg. Psychiatry.

[18] Tian C., Cao X., Wang J. (2017). Recanalisation therapy in patients with acute ischaemic stroke caused by large artery occlusion: Choice of therapeutic strategy according to underlying aetiological mechanism?. Stroke Vasc. Neurol..

[19] Kim B.M. (2017). Causes and solutions of endovascular treatment failure. J. Stroke.

[20] Kim S.K., Baek B.H., Lee Y., Yoon W. (2017). Clinical implications of CT hyperdense artery sign in patients with acute middle cerebral artery occlusion in the era of modern mechanical thrombectomy. J. Neurol..

[21] McVerry F., Liebeskind D., Muir K.W. (2012). Systematic review of methods for assessing leptomeningeal collateral flow. AJNR Am. J. Neuroradiol..

[22] Kim B., Chung J., Park H.-K., Kim J.Y., Yang M.-H., Han M.-K., Jeong C., Hwang G., Kwon O.-K., Bae H.-J. (2017). CT angiography of collateral vessels and outcomes in endovascular-treated acute ischemic stroke patients. J. Clin. Neurol..

