# Supplementary material for: Utility of Leptomeningeal Collaterals in Predicting Intracranial Atherosclerosis-Related Large Vessel Occlusion in Endovascular Treatment
Source: J Clin Med. 2020 Aug 28;9(9):2784. doi: 10.3390/jcm9092784 (PMC7564225; doi:10.3390/jcm9092784)
Supplement: Supplementary file 1 [file jcm-09-02784-s001.pdf]

SUPPLEMENTARY MATERIAL

Table S1. Multivariable analyses for the association with intracranial atherosclerosis-related large vessel occlusion

	Model 1		Model 2		Model 3		Model 4	
	Odds ratio (95% CI)	*P*-value	Odds ratio (95% CI)	*P*-value	Odds ratio (95% CI)	*P*-value	Odds ratio (95% CI)	*P*-value
Current smoking	3.08 (1.32–7.18)	0.010	3.08 (1.32–7.18)	0.009	2.82 (1.24–6.43)	0.014	3.06 (1.31–7.14)	0.010
Atrial fibrillation	0.45 (0.20–1.01)	0.053	0.44 (0.20–0.99)	0.048	0.43 (0.19–0.96)	0.040	0.44 (0.20–0.99)	0.047
Initial NIHSS score	0.93 (0.86–1.01)	0.069	0.93 (0.86–1.00)	0.061	0.92 (0.85–0.99)	0.031	0.93 (0.86–1.00)	0.055
Leptomeningeal collaterals								
Three assessment methods								
Method 1 *(Tan)*								
0%	Reference							
> 0% but ≤ 50%	1.40 (0.14–13.9)	0.774						
> 50% but < 100%	1.58 (0.17–14.3)	0.685						
100%	4.94 (0.55–44.5)	0.155						
Method 2 *(shortened Maas)*								
0%			Reference					
> 0% but < 100%			1.51 (0.17–13.2)	0.708				
100%			4.91 (0.54–44.3)	0.157				
Method 3 *(modified Tan)*								
≤ 50%					Reference			
> 50%					1.99 (0.74–5.31)	0.170		
New method								
Incomplete (< 100%)							Reference	
Complete (100%)							3.32 (1.52–7.26)	0.003

CI, confidence interval; NIHSS, National Institutes of Health Stroke Scale.
